# The obscured face in video consultations. A Levinasian analysis

**DOI:** 10.1007/s11019-025-10271-w

**Published:** 2025-04-26

**Authors:** Elisabeth Assing Hvidt, Frida Greek Kofod, Johannes van den Heuvel, Michael Scheffmann-Petersen

**Affiliations:** 1 Research Unit of General Practice, Department of Public Health, University of Southern Denmark, Campusvej 55, 5230 Odense M, Denmark; 2 Research Unit for General Practice, Department of Public Health, University of Copenhagen, Copenhagen, Denmark; 3 Department of Clinical Medicine, Center for General Practice, Aalborg University, Aalborg, Region Nordjylland Denmark; 4 Primary & eHealth Care, Region Zealand, Department of Communication & Arts, Roskilde University, Roskilde, Denmark

**Keywords:** Video consultations, General practice, Phenomenology, Levinas, Buber, Ethical Telecare

## Abstract

Video consultations represent a relatively new way of delivering face-to-face consultation in the context of general practice. The aim of the present analysis is to examine how video consultations influence patients’ experiences of their ability to communicate their emotions, needs, and vulnerabilities, as well as their experiences with their general practitioner’s ability to respond to these. The empirical base consists of 43 semi-structured interviews with patients (23 women and 20 men), aged between 17 and 81 years old, who have used video consultation as part of their treatment for various health issues in general practice. Emmanuel Levinas’ theory of the face was used as an analytical and interpretative tool. The analysis showed that in video consultations, patients experience a digital obscuring of the face, i.e., of their emotions, needs and vulnerabilities. This complicates the GP’s ability to perceive their vulnerability, making it challenging to recognize the patients’ needs. Moreover, this obscuration hinders the patients’ capacity to connect to their own vulnerabilities, which can lead to a diminished awareness of their own suffering. However, this mechanism might help patients who wish to obtain shielding from their face and from difficult emotions. Overall, we argue that significant relational and ethical dimensions of care within the doctor-patient relationship in general practice may be challenged in video consultations. We propose integrating Buber’s dialogical principles with the present Levinasian analysis as it may offer a promising approach to enhancing relational and ethical dynamics in video consultations.

## Introduction

Digital consultations, mediated through telephone, email or video, have increasingly become part of our common experience of receiving healthcare within many Western general practice settings (Assing Hvidt et al. 2023; Atherton et al. 2018; Greenhalgh and Rosen 2021). While telephone and email consultations have gained (more or less) stable ground, video consultations, introduced in the context of the COVID-19 pandemic when in-person care was restricted, represent a relatively new way of delivering face-to-face consultation in general practice (Assing Hvidt et al. 2023).

The synchronous nature of video consultations, combined with modern technology’s visual and audible conditions (with very low time lags), has led scholars to explore whether video consultations can facilitate health care quality equivalent to in-clinic face-to-face consultations (and superior to telephone consultations), potentially serving as a viable alternative to in-clinic face-to-face consultations (Atherton et al. 2018; Donaghy et al. 2019; Stieger et al. 2023). Studies conducted within a general practice setting show that if used appropriately, video consultations seldomly lead to missing out on symptoms (Johnsen et al. 2021; Wanderås et al. 2023) and that they despite the physical distance may create a sense of being present with the general practitioner (GP), an experience known in communication theory as “mediated presence” (Lee 2004).

This has led scholars to conclude that video consultations are suitable for health issues that do not require direct physical examination, for example, issues that can be easily assessed visually or managed through talking, including psychotherapeutic treatment (e.g., talking therapy) in connection with patients’ mental health problems (Donaghy et al. 2019; Wanderås et al. 2023).

However, challenging the above conclusion, other research on communication in video consultations in general practice brings attention to how video consultations alter perception and interaction modes, potentially impacting the communicative and interactional quality of the patient-doctor encounter (Jepsen et al. 2022; Kofod et al. 2024a). For example, video consultations have been found to reduce or even eliminate social talk, shared decision-making processes and patients’ “door-knob” questions, i.e., questions asked by patients that reveal significant and personal information just as the consultation is about to end (Nielsen 2012). Studies examining patients’ and clinicians’ experiences with video consultations consistently reveal that despite the benefits of increased access, convenience, and time efficiency, both parties feel that certain fundamental interactional features are missing (Assing Hvidt et al. 2022; Due et al. 2021; Nordtug et al. 2022; Van den Heuvel et al. 2024). A recent study conducted in Danish general practice by Kofod et al. (2024b) revealed that GPs experience a diminished sense of their patients during video consultations as well as reduced presence due to lacking co-spatiality. As a consequence, and to achieve “good enough” interpersonal care, they try to compensate by modifying their communicative verbal and nonverbal behavior (Kofod et al. 2024a). Additionally, they avoid video consultations with patients with so-called complex issues and with whom they have not an antecedent relationship.

Outside of a general practice context, within the fields of psychotherapy and psychiatry where health care provision rarely requires physical examination, video consultations play a large role and become increasingly embedded into daily clinical practice. Empirical research indicates positive effects on measurable outcomes such as patient satisfaction and clinical outcomes (Sharma and Devan 2023). Some studies, however, have focused on the less measurable moral and ethical dimensions of telepsychiatry (Bizzari 2022; Frittgen and Haltaufderheide 2022; van Wynsberghe and Gastmans 2009). These studies problematize the ability to establish therapeutic alliance, rapport and resonance with patients within the context of technology-mediated therapeutic interactions. Providing an exhaustive review of video psychotherapy and psychiatry, however, is beyond the confines of this paper.

The research findings mentioned above raise the question of how video consultations alter the relational and ethical dimensions of the doctor-patient relationship in general practice.

Investigating this question is a way to ensure not only medically safe video consultation practices but also those that are relationally and ethically responsible and responsive.

From a medical humanities and bioethical perspective, the relationship between a doctor and a patient is considered an ethical interaction that is distinct from, for example, a commercial transaction. Bioethicist Pellegrino (2006) argues that the physician-patient relationship is a healing relationship, rooted in the patient’s vulnerability and request for aid, coupled with the doctor’s medical expertise and commitment to help. A sound medical decision is based on both the physician’s medical knowledge and an empathetic understanding of the patients’ illness experience and lifeworld. Writing before the emergence of online clinical consultations, Pellegrino’s insights into the healing relationship are grounded in the physical interactions between patients and doctors characterized by the possibility to observe the patient’s physical expressivity.

Within the contemporary phenomenological tradition, scholars have investigated the nature of communication in online encounters (Carel 2020; Dolezal 2020; Fuchs 2014; Grīnfelde 2022; Osler 2020). Some of them argue, that due to lacking physical proximity in online face-to-face interactions, significant features of intercorporeality^1^, empathy and interaffectivity^2^ are missing (Carel 2020; Dolezal 2020; Fuchs 2014).

Dolezal, drawing on Hubert Dreyfus (who builds on Emmanuel Levinas’ ethics), argues that the most important feature missing in online encounters is the experience of the other person’s emotional and existential vulnerability that might affectively move us and guide our ethical response towards that person (Dolezal 2020). Consequently, online encounters are less real and less authentic than fully embodied encounters. In a similar vein, Carel (2020) argues that digital encounters are a “poor replacement for embodied engagement with others” (p. 12). An even more radical stance is the one taken by Fuchs (2014) who argues that online communication is “non-sensous” (p. 167) and, consequently, disembodied. In other words, all of the above-mentioned phenomenologists agree that in online encounters important social and affective cues are reduced and, consequently, also the possibilities for empathetic perception of the other’s experiential states, such as feeling emotionally and existentially vulnerable.

Representing a different view, Osler (2020) argue that despite the lacking physical proximity and reduced perceptual richness in online encounters, new ways are stimulated in which people act and interact, potentially creating moments of intense “we-experiences” (e.g., in encounters on social platforms). Continuing this line of thought, Osler and Zahavi (2022) argue for a more nuanced approach to understanding and assessing online forms of sociality. Digital encounters are inherently different, constitute their own forms of sociality and require an analysis that goes beyond a simple replacement framework.

Aligning with the recognition that online communication has unique characteristics and must be analysed phenomenologically on its own terms, Lindemann and Schünemann (2020) propose that combining the phenomenological concepts of the lived body and mediated immediacy from Helmuth Plessner with Hermann Schmitz’s concept of embodied space can help “depathologize” digital communication. Similarly, Burow (2022), focusing on video-mediated interaction, suggests that participants actively create “Leiberspace” in the context of digital communication. This term does not refer to a physical location or space but describes the state and process of shared bodily experiences in digitally mediated interactions.

Within the context of teleconsultations within specialized medical care, it is argued by Grīnfelde (2023) that physical proximity is not a necessary condition for either empathetic or ethical provider-patient relationships and that teleconsultations might even lead to more open, relaxed and patient-centred clinical encounters. While the above research sheds light on the possibilities of empathetic perception and presence in online encounters, including clinical consultations, there is still limited knowledge regarding how patients experience that video consultations influence the ethical dimension in the communication with their GP, particularly in expressing emotional depth and inherent vulnerabilities.

The aim of the present paper is thus to analyse how video consultations influence patients’ experiences and perceptions of their ability to communicate their emotions, needs, and vulnerabilities, as well as their experiences with their GP’s ability to respond to these. This aim differs from the approaches taken in the above-mentioned research by combining a focus on emotional and vulnerable aspects of communication in the specific context of video consultations in general practice with a patient-centred perspective. It goes beyond assessing general satisfaction and the nature of patient-provider communication in online communication to explore the nuanced relational and ethical dynamics in video consultations in a general practice setting characterized by a commitment to establishing strong doctor-patient relationships through continuity of care (Ladds et al. 2023).

Emmanuel Levinas’ (1987, 1991) ethical philosophy, focusing on how we relate and respond to one another in an ethically responsible manner through the face, is useful for exploring ethical relationality in a context that - while lacking direct, embodied face-to-face interaction - still involves technology-mediated perception, experience and sociality (Lee 2004). Thus, in the context of video consultations, emotions, needs and vulnerabilities may still be communicated through the technologically mediated face. For example, and building on the above phenomenological research, the face of the other, mediated through the screen, may still convey aspects of the face in a metaphorical sense, eliciting an ethical response to the other that transcends spatiality. However, to which extent this is happening remains unexplored.

### Emmanuel Levinas’ ethics

Levinas’ phenomenological ethics focuses on the face-to-face encounter with the other in the everyday social life. This encounter is a fundamental ethical event in which the other’s vulnerability and humanity present themselves to the self (Morgan 2011, p. 59).

During daily life experiences, social relationships are characterized by reciprocity and etiquette: we are caring towards one another, we reciprocate other persons’ favors, angers, injustices, etc. Below this surface of reciprocity, Levinas says, lies a non-reciprocal dimension where the other confronts the I as complete other, in dependence, thus presenting a vulnerability. At this deep ethical layer in the encounter, hidden in every ordinary social relationship, the I is confronted with an absolute choice: on a fundamental and abstract level, to let the other live or die; to support his/her living or death (Morgan 2011, p. 71). On a less abstract level, representing a basic layer of relationality and vulnerability, the other is confronted with a sense of dependence through the face of the other that at the same time demands to be treated with respect, concern and care. Levinas (1987) writes: *The epiphany of the absolutely other is a face*,* in which the other calls on me and signifies an order to me through his nudity*,* his denuding. His presence is a summons to answer* (p. 97).

Levinas emphasizes that the other, confronting me through the face, is fundamentally distinct from the I, what the I is not (alterity), and thus beyond any classification and categorization. The face is thus the responsibility to acknowledge and accept the other, rejecting objectification and instrumentalization. Levinas distinguishes between totality and infinity, where the former concept reduces the latter based on my expectations and classifications. The concept of infinity, on the contrary, is about everything that we cannot understand about the other, everything that is different from ourselves. In Levinas’ perspective, the human’s face and eye contact are an image of the infinite, which means that we cannot meet the other from a totalitarian perspective since the other presents a perspective that can never be accessed directly. Levinas (1991) writes:

The face is present in its refusal to be contained. In this sense it cannot be comprehended, that is, encompassed. It is neither seen nor touched - for in visual or tactile sensation the identity of the I envelops the alterity of the object, which becomes precisely a content (p. 194).

Realizing this, means the end of mastery of the other. In other words, it is the encounter with the face and eyes of the other that opens an appeal for care in the relationship. Why did Levinas choose the face as notion to indicate this relational and ethical responsibility in the encounter with the other person? Morgan (2011) explains that Levinas chose this because the face is characteristic of a living being, with both its bodily and metaphysical presence. The face is the most revealing physical expression of a person’s inner self, reflecting their feelings, thoughts and attitudes. It clearly conveys their needs, pain, joy, sorrow, and suffering. Notably, the eyes provide a window into how someone is truly feeling. However, Levinas emphasizes that the face of the other is not merely the appearance of that person; it is not a collection of features given to visual perception. The face holds profound metaphysical significance and is a metaphor for the encounter with the other presenting an inescapable ethical demand to not do harm and take responsibility for the other person. This demand can also present itself to me indirectly through cognition, signifying an imposing presence. Thus, although, in Levinas’ ethics, the responsibility to the other, not least through the gaze, is highly related to physical proximity, we contend that Levinas’ notion of the face, and the fundamental tenets underlying his ethics, applies to both online and offline contexts.

We furthermore argue that Levinas’ philosophical views on the other, the face-to-face encounter, ethical responsibility and vulnerability can help us think more deeply and richly about how patient experience relationality and vulnerability in video consultations.

## Methods

### The empirical setting

General practice in Denmark is the citizen’s first point of contact with the healthcare system. All citizens have free access to general practice and are registered with a GP of their choice within (ideally) their geographical proximity (Nexøe 2013; Pedersen et al. 2012). General practice handles a wide range of health issues - from chronic diseases and mental challenges to acute conditions. Following a consultation, the GP either continues or concludes the treatment within general practice or refers the patient to specialized care in other parts of the healthcare system. Patients can consult their GP or the practice staff in person (clinic visits, constituting the vast majority of consultations), by telephone, through written e-consultations or video consultations. Introduced in connection with the Covid-19 pandemic, video consultations have since 2025 become mandatory for all GPs to offer as a political push towards increased efficiency, resource optimization and patient flexibility and empowerment (Regeringen and Danske Regioner 2022). Patients can either access video consultations through a freely available and secure application or by clicking a sms link sent to them by their GP.

Video consultations have had a slow uptake in general practice in Denmark, currently only accounting for 1.4% of all consultations in general practice. Traditionally, the GP in Denmark is regarded as the patient’s “own doctor”, meaning that patients, whenever possible, are treated by the same GP. This approach supports quality principles related to person-centered care, continuity of care, and multidimensional care that requires a holistic assessment of the patient’s health problems. However, general practice has in recent years undergone many changes and transformations due to requirements for adapting to challenges and innovations in the health care sector and in society at large (e.g., a changed demography, technological advancements, policy and reform, shifting care models and workforce challenges) (Ladds et al. 2023; Simonsen et al. 2024). This implies that “old ways” and ideals in respect to doctoring are being challenged.

### The empirical project

The data used in this study was collected as part of a large qualitative research project investigatingthe implementation of video consultation in Danish general practice. Parts of this research focused on the relational, interpersonal and communicative aspects of the doctor-patient relationship in this online context. While some of the research capture the GPs’ perspective, the present paper represent the patient perspective only.

The data corpus consisted of 43 semi-structured interviews with patients (23 women and 20 men), aged between 17 and 81 years old who had experiences with video consultation as part of their treatment for various health issues in general practice. We attempted to apply a purposive sampling method, ensuring variation in patients’ sex, age, geographical location, size of the clinic and volume of video consultation use. To recruit patients with experience with VC in general practice, we used two approaches. One approach was GP-mediated recruitment. We contacted from regional lists by telephone and reached out to GPs in our professional network to ask for their assistance in inviting patients to participate in the study. The other approach was direct-to-patient outreach. We posted an advertisement on social media platforms to invite patients to participate. The majority of patients were recruited via the GPs and were connected to varying clinic sizes and living in three out of Denmark’s five regions. Via the social media recruitment approach we recruited patients from varying clinic sizes and living in two out of Denmark’s five regions. The interviews were conducted from November 2021 to March 2024 by various interviewers: the authors [anonymised] and [anonymised] and by three other researchers (one former PhD student, one PhD student and a former graduate student). The research aims and procedure were introduced to the patients before each interview and all patients gave written consent and were informed that participation in the study was voluntary. All sub studies received approval from the research institutions’ institutional boards and ethical committees (approval numbers [anonymised]).

Depending on the patients’ preference, interviews were either conducted online (*n* = 11) through the secure Zoom and/or Teams platforms, by telephone (*n* = 22) or face-to-face (*n* = 10). Semi-structured interview guides were used including open-ended questions, focusing on the experiences with communication and interpersonal contact between the patients and the GPs in video consultations. Comparison between experiences deriving from video consultations and in-clinic physical consultations came up naturally in the interview context. All interviews were conducted in the Danish language, transcribed verbatim and pseudonymized. Selected data for the purpose of this article were translated by the authors. In a first analytic phase, the interview data were coded inductively by the authors [anonymised] independently, drawing on the analytic principles of either thematic analysis (TA) (Braun and Clarke 2006) or interpretative phenomenological analysis (IPA) (Smith et al. 2009). Discussing the codes and themes collectively, and inspired by an abductive analytic approach (Timmermans and Tavory 2022), the first and second author identified a provisional ‘theoretical case’ related to Levinas’ ethics and face-to-face relationships. This was discussed with the other authors who tested the theory up against their data, finding that Levinas’ theory of the face “rang true” and could be adequately used as analytical and interpretative tool. Analytic discussions going back and forth between the empirical interview material, the codes and Levinas’ theory subsequently led to the following three themes: (1) The face becomes obscured during video consultations, (2) The ethical responsibility of the GP becomes obscured, (3) The connection to one’s own inner self becomes obscured during video consultations.

## Findings

### The face becomes obscured during video consultations

In the first part of the analysis, we draw on Levinas’ understanding of how human emotions, needs and vulnerabilities manifest through the face which in turn calls the other person to respond (Levinas 1987). We argue that beneath the surface level of being able to see one another in video consultations lies an experience of an incomplete interpersonal understanding and human contact that results from what we call the obscuring of the face. In the following, we shall substantiate this argument with examples from our data material.

Jack, a 27-year-old man diagnosed with chronic depression and anxiety, conditions he has been managing with treatment for the past ten years, notes that despite video consultations allowing for eye contact, which is nice “when talking about…how can I put it…some slightly vulnerable things”, they fall short of in-clinic face-to-face consultations. He emphasizes that when meeting physically “you see each other more”. From the conversation with Jack, it is clear that he believes that access to full body language during in-clinic face-to-face consultations is crucial for a comprehensive understanding of the other person.

Eileen, a 31-year-old woman receiving treatment in general practice for stress and depression, also reflects on the experiential differences between her in-clinic face-to-face consultations that she had pre-pandemic and video consultations that she had during the pandemic (due to lock-down measurements). Eileen felt that her psychological symptoms were more visible to her GP during in-clinic face-to-face consultations, allowing her GP to grasp her vulnerability on a deeper level. In contrast, during video consultations, she felt that the lack of nuanced body language made it difficult for her GP to fully understand her emotional state and the extent of her distress: “When it was in the clinic, I somehow felt that it was visible that I was feeling bad. You know, that thing… the fact that the psychological can actually be seen physically… it was also a form of vulnerability in a way, right?”

Like Jack, Eileen touches upon the paradox of video consultations: while they allow for visual perception, they reduce the ability to sense the other person’s vulnerability that is not directly visually perceptible. The above experiences align with Levinasian themes. The face is more than just appearance given to visual perception. It reveals the other person’s vulnerability and is thus about presence, recognizing and responding to the other person’s needs.

Referring to what we have termed the obscuring of the face in video consultations, several participants discussed the importance of physical proximity at the onset of treatment, particularly when the health issue involved vulnerable matters. For example, Eileen expressed satisfaction that her personal treatment trajectory had not started with video consultations, even though she appreciated that video consultations eliminated the stress of transportation and waiting rooms. For Eileen, it was not only a question of the GP seeing her face but also a question of her “getting a sense of who she (the GP) is as a person. And this is important in such a vulnerable trajectory”.

Likewise, Jack noted that he would not have been comfortable consulting his GP through video consultations ten years ago when he felt more vulnerable. He stressed that for someone living in more precarious situations due to mental illness video consultations might not be suitable. Touching on the same point, Mette, a 33-year-old woman in treatment for anxiety and depression in general practice, said:

I would never… if I were coming in completely new with some kind of psychological issue, I don’t think I would choose a video consultation. I would need to look my doctor in the eyes for real. So they could also sense who I am. I just think I would feel that I… I would need to go to the doctor and sit across from them, so they could really understand how distressed I was—or am… it’s about needing to look a person in the eyes. I need to show my vulnerability… and I feel that gets lost through a screen.

The above accounts, we argue, exemplify experiences of an obscured face in video consultations. As a result, a relational and hermeneutic dimension is missing. This dimension includes being able to present oneself to the other as a whole person with emotions, needs and vulnerabilities, and the other sensing that responding to this humanness is an ethical responsibility. In the second part of the analysis below, we argue that the obscuring of the face obscures the deep-layer ethical responsibility of the other—in our case, of the GP.

### The deeper ethical responsibility becomes obscured during video consultations

As mentioned above, Levinas’ ethics focuses on the face calling the other person to respond (Levinas 1987). This implies that we are always already ethically responsible to the other person we meet through the face (Morgan 2011). We claim, based on the interview material, that the GP’s relational and ethical responsibility towards the patient becomes obscured during video consultations due to the face being obscured. We also claim that this obscuration makes the video consultation encounter more transactional and focused on the specific reason for the encounter. We shall substantiate this argument in the following with examples from our data.

In our interview material, some patients characterized video consultations as more rushed than in-clinic face-to-face consultations. For instance, Mette described video consultations as encounters where “time is short” and that they are completed as “pearls on a line”, wanting to evoke the idea of something being done in an efficient manner. Jack, having video consultations with his GP every third month, primarily to check his medication dosage, characterized his video consultations as both rushed and pre-structured: “It’s very much like… we just need to get through these questions… and then we’ll quickly find a conclusion…like: “What do we do? Should we go up, should you go down—get a new prescription… see you, take care, bye”. Jack contrasted this experience with encounters with his GP in the clinic, saying:

When I’m physically at the doctor’s, it doesn’t have to be over as quickly. I’m not just rushed through some questions, and then we say ‘bye’… but if I had come to him physically with an ingrown toenail… he probably would have also asked ‘How are you doing?’ And then I might have thought ‘Well, you know what, I’ve actually been arguing a bit with my girlfriend lately’. And then he would say ‘Oh… what do you say to having a conversation soon about how things are going? You can just book a new appointment’.

As Jack suggests in the above excerpt, the layer of human relating where the GP shows interest in the patient’s overall well-being, beyond what is specifically dealt with as the reason for contact, is missing in video consultations. Drawing on his experiences with face-to-face encounters with his GP, he imagines an alternative interactional scenario within a face-to-face encounter in which the GP asks him how is doing, empathetically encouraging him to become vulnerable and responding to his answer by giving him the opportunity to talk more about it in a later encounter.

Seen from a Levinasian perspective, the example given by Jack suggests that only once the GP is confronted with the face in the in-clinic face-to-face encounter can he sense his appeal, feeling responsible towards him and addressing him as a person with needs beyond the health issue he is seeking help for. In this instance with Jack, his actual need may manifest as a desire to share his lifeworld with his GP. Consequently, the video consultation as a mode of communication obscures a deep layer of ethical responsibility, making it difficult for the GP to decode and sense the patient’s vulnerability, or call for help, and to react in an ethically responsible manner.

A related point regarding the difficulty of sensing and reacting to the other person’s needs within an interactional context of video consultations was touched upon by Steven, a 68-year-old participant who had consulted his GP over video for non-complex physical symptoms. He said:

It’s a slightly more impersonal experience when it’s over video. Uh… it’s really… it’s my intuition, that… I mean, my experience of how we… the other person… how we react to each other, that’s different when it’s over video. It’s more… it’s more exchanges of opinions… I mean, you can have something over video that resembles something emotional that we can discuss. But, but still… my experience… my own personal experience of it is that there’s something missing in terms of depth. That’s the closest I can get… using that word [depth] for it.

In the above excerpt, Steven finds it difficult to put into words what is missing in the interpersonal exchange in video consultations. Eventhough something akin to emotional interaction can occur, he says, this still entails a transformed - and less profound - mode of interaction. Relating to Levinas’ philosophy of the face, it is plausible to suggest that the missing element in video consultations is the speaking face that represents a deep layer of ethical connection, and that according to Levinas lies beneath the surface of any social everyday interaction. Steven seems to suggest that in the relationship enacted through a video consultation this deep layer of interaction is obscured, which renders the encounter more transactional and less meaningful. To compensate for the missing profoundness in the interaction, Steven maintains the importance of supplementing video consultations with in-clinic face-to-face encounters. He elaborates: “In a way, I still prefer that we also meet in person. Because there are, after all… uh… several different things we will end up talking about.” Expressed by Steven in the above excerpt is also the experience that in-clinic face-to-face encounters opens to talking about various subjects, indicating that the in-clinic face-to-face encounter is experienced as an encounter in which the GP is to a higher degree than in video consultations open and sensitive to responding to whatever the patient senses a need for expressing, as also seen in Jack’s case. Delving into Levinas’ ideas about totality and infinity (Levinas 1991), it can be argued that from a patient perspective the in-clinic face-to-face encounter offers an interactional context in which favorable conditions exist for allowing the patients to develop and share their vulnerability within an ethical relationship, that is, a relationship in which the other is not objectified, classified or categorized. In the third part of the analysis, we demonstrate that when the face and the GP’s ethical responsbiblity is obscured, it obscures the patient’s ability to sense their own emotions, needs and vulnerabilities, which, in turn, highlights an important relational dimension.

### The connection to one’s own inner self becomes obscured during video consultations

According to Levinas, encountering the face of another person allows one to connect with one’s own inner self, feelings and vulnerabilities. In the eyes of the other, one becomes present to oneself and to one’s needs, pain, joy, sorrow and suffering (Morgan 2011). Based on the interview material, we claim that the obscuring of the face - and the ethical responsibility it invokes—results in an obscuring of the connection to one’s own inner self during video consultations. We shall substantiate this argument in the following with exemplary data extracts.

Mette, also cited above, emphasized that perceiving the full presence of her GP in in-clinic face-to-face consultations would enhance her ability for expressing herself:

…especially when it comes to psychological issues, I just think it’s incredibly important for me to feel the other person I’m sitting across from. I find it easier to express myself if I’m physically sitting across from a person, rather than looking into a screen.

Like Mette, another participant, Bodil, a 68-year-old woman suffering from anxiety and depression, experienced diminished abilities to “open up” during video consultations: “I don’t feel that I can open up in the same way in a video consultation. It removes me because I lose my grounding”. Bodil, in addition to losing her own grounding in video consultations, also commented on a perceived lacking presence of her GP in video consultations which, she noted, might be connected to her own lacking presence.

I actually also think that she [the GP] seems more absent—or maybe it’s me who is more absent. That could also be the case. I like it when people show presence, and it probably slips a bit in a video consultation—at least for me.

Thus, for Bodil, video consultations not only hindered the sense of co-presence with her GP, but also acted as a barrier to her own presence. As a result, video consultation was perceived as a source of alienation. In the above excerpts, we observe a relational dimension and effect that aligns with Levinas’ thoughts on interpersonal connections. The digital obscuring of the face not only complicates our ability to sense vulnerability in the other (and their call for help) but also hinders our capacity to connect to and acknowledge our own vulnerabilities. The consequence of not connecting with vulnerabilities in digital meetings (unless explicitly agreed upon) often results in digital meetings being perceived as more superficial, absent, and distracting. This was voiced by Cathrine, a 25 year old student, who consulted her GP for depression and eating disorder:

I think I become more superficial. I don’t think I get as close to my feelings when I talk [on] video as I usually do. I don’t know—I just think that when you’re sitting across from a person, physically, it’s just so natural for me to dare to share myself if I feel safe in the other person’s hands.

Eileen, in addition to emphasizing the superficiality of video consultations, described her own behavior during video consultations as less spontaneous compared to in-clinic face-to-face interactions. As a result, Eileen refrained from sharing details about her well-being during video consultations. She concluded: “there is like some conversations that are not occurring at any rate. Because I don’t get into contact with that part of me or she [the GP] doesn’t see it, or…” Altogether, the above experiences emphasize a relational ethics whereby the understanding of ourselves as subjects and, for example, our understanding of the root of our mental suffering, is formed and dependent on other subjects who through the face (presence and attunement), awakens and brings it into being.

While the excerpts above illustrate the downsides of obscuring the face—along with the ethical responsilbity that it invokes and its impact on connecting to one’s inner self - this obscuration can also serve as a mechanism for self-protection and self-control. For instance, 24-year-old Caroline reflected on a period during the pandemic when she struggled with mental health issues. She felt a need to shield herself from the empathy her expressivity might provoke in her GP during a face-to-face encounter. As she explains, her GP’s empathic reaction could lead her to break down emotionally, which she did not feel ready to handle:

I felt really bad during the corona period, with anxiety and such. And I also thought it was a bit like, should this be done over video, or should it not be done over video. […] The thing is, when you feel that they actually see and hear what you’re saying and trying to say [in an in-clinic face-to-face consultation], it can make you start to break down. Because finally, there’s someone who actually listens to what you have to say. Like… Yeah. And maybe I’m not so good at that. I might prefer that no one really knows.

The above excerpt highlights some perceived benefits tied to the obscuring of the face in video consultations, exemplifying how patients perceive the effects of video consultations differently depending on their individual needs. For Caroline, the obscuring of the face in video consultations helps shield her face, including her vulnerability, from the GP and from herself. Moreover, the above excerpt suggests that it prevents this vulnerability from becoming a shared experience between her and the GP which, altogether, puts her more at ease because she does not have to think about how to manage potential difficult feelings. Thus, on a more general note, the obscuring of the face in video consultations provides patients who want to maintain their emotional boundaries between themselves and their GP with more control over their vulnerability disclosure.

## Discussion

In this study we have explored how video consultations influence patients’ experiences and perceptions of their ability to communicate their emotions, needs, and vulnerabilities in the context of the doctor-patient relationship, as well as their experiences of the GPs’ ability to respond to these. To that end we have been drawing on Levinas’ phenomenological ethics and concept of the face. Applying Levinasian ethics to video consultations implies moving beyond the question of whether video consultation *can* be used (focusing on feasibility and acceptability) to the question of the ethical and relational implications of video consultation use. Through our analysis of patient experiences, we have identified three interrelated mechanisms that structure patients’ experiences, providing insight into the relational and ethical significance and challenges of video consultations as they are currently practiced in Danish general practice. First, the digital mediation in video consultations obscures the face, implying that the patient’s emotions, needs and vulnerabilities communicated through the face are not fully communicated and hence not fully grasped by the GP through the screen. Second, this obscuration of the face directly impacts the GP’s sense of ethical responsibility towards the patient. Put differently, once the face of the patient, acting as a powerful ethical signifier, is obscured, the ethical responsibility towards the individual is likewise obscured. This potentially compromises the depth of ethical engagement with the patient. Third, the experiences of diminished co-presence in video consultations may obscure patients’ connection to their inner selves. Aligning with Levinas’ thinking, this mechanism reveals a relational ontology whereby the self is awakened in the encounter with the other and upon the other’s response. When a digital obscuration of the face occurs, patients’ ability to fully connect with their own experiences and needs during the virtual consultations is challenged which, in turn, may further complicate the ethical dynamics of the virtual doctor-patient relationship. For some patients, however, this precise mechanism helps them obtain a desired shielding from their face and from difficult emotions. This resonates with existing research showing that some patients dealing with psychiatric issues prefer using video consultation for treatment purposes because the digital distance makes it easier to discuss and open up to sensitive topics (Bizzari 2022; Sharma and Devan 2023).

Seen from a Levinasian perspective, the above mechanisms collectively challenge fundamental ethical processes of relating in the context of virtual consultation, leaving room for an environment conducive to more transactional and objectifying encounters. Even though video consultations can represent good health care and even empathic interaction, our results suggest that when what is at stake is the therapeutic process and mechanisms linked to it, including ethically and holistically felt vulnerabilities, video consultations can be less fitting.

Supporting this perspective, Moore et al. (2024), describe how video consultations alter embodiment, perception and the clinical gaze, ultimately changing “what is seen and known” (p. 420). As a result, both patients may feel that the experience of receiving care has been disrupted, based on a feeling that they have not been really “seen” or properly treated. The authors emphasize that “video-consulting is not a straight forward replacement of in-person consulting” (p. 430), underscoring the need for a critical examination of how social and ethical processes of relating are facilitated by users.

Importantly, and countering the above-mentioned essentialist take of some phenomenologists on online encounters as disembodied, laking interaffectivity, ethical responding, etc., we do not assert that physical meetings guarantee ethical interactions and conduit. As noted by others (e.g., (Lindemann and Schünemann 2020), we can be physically present to one another, yet socially, psychologically and emotionally absent. Furthermore, we contend that there is nothing inherently relational or ethical about the in-clinic face-to-face consultation as if this meeting carries *intrinsic* value. Instead, both online and offline interactions offer opportunities for connection and disconnection with others—and for revelation or obscuration of the face.

Reflecting on how to enhance relational and ethical dynamics in Denmark’s (and other Western countries’) evolving digital healthcare landscape, we propose contextuliazing our Levinasian analysis, and the challenges it highlighs in digital synchronous interactions, with Martin Buber’s understanding of symmetrical dialogical encounters. Buber, like Levinas, was a significant 20th century thinker who profoundly contributed to our understanding of intersubjective encounters (Meindl et al. 2020). Both philosophers recognize that encounters with others extend beyond mere logical and cognitive interaction. However, they approach intersubjectivity differently. Whereas Levinas emphasizes the face, highlighting unconditional and non-reciprocial ethical responsibility for the other, Buber conceptualizes the dialogical meeting as relying on reciprocity. Buber distinguished between two types of relationships: *I-It* relationships characterized by an instrumental approach where one experiences the other unilaterally as a passive object and *I-Thou* relationships defined by mutual awareness and presence that encourages horizontal dialogue (Buber 1970, 2003). According to Buber, in *I-It* relationships, one experiences and grasps the other at a surface level and within an active-passive divide. The other offers no resistance to the grasping subject. In *I-Thou* relationships, both parties are reaching out to the other, aiming at establishing a common sphere in which feelings, perceptions, needs and thoughts are made present to one another (Meindl et al. 2020).

We argue that Buber’s focus on dialogical relationships can complement Levinas’ ethical framework, encouraging a more symmetrical dialogical approach to the other in the video consultation, particularly in the context of addressing vulnerabilities. To unmask what might be obscured in video consultations, both patient and GP must commit to a form of dialogical engagement that resembles Buber’s *I-Thou* relationships. This approach can help counteract the potential distancing effect of video technology by fostering mutual awareness and presence. It should be noted that this approach does not negate the inherent asymmetry within the doctor-patient relationship nor Levinas’ emphasis on ethical responsibility. Rather, it suggests that striving to overcome the challenges in digitally mediated interactions, and to promote the ethical processes of relating that Levinas advocates, a focus on more reciprocal dialogue might be necessary.

We believe that the way video consultations are practiced and experienced, by GPs and patients, is inseparable from, and indeed intertwined with, a broader socio-cultural and political context. This context ties the digitalization of healthcare to logics driven by acceleration and efficiency (Simonsen et al. 2024), fostering, it can be argued, *I-it* relationships. Proactively approaching video consultations as modes of communication that can facilitate the revelation of the face (as opposed to obscuration), sparking dialogue about vulnerabilities, is thus essential for promoting “the art of medicine” (Borsch et al. 2024) within an increasingly complex, partly digital, consultation ecology. Furthermore, this approach challenges stereotypical and deterministic narratives that depict technologies as leading to the provision of “cold” or “warm” healthcare (Lüchau et al. 2024; Pols 2012).

Limitations.

This study has several limitations. First, our study represents the patient perspective only. Investigating the inter-relationship between patients’ and GPs’ experiences, and how they might be in tension or alignment, could thus have strengthened the analysis. This limitation has several implications as it may present an incomplete picture of the consultation dynamic, missing crucial insights into the reciprocal nature of the doctor-patient interaction during video consultations. GPs’ perspectives could have revealed challenges or benefits that patients might not be aware of or able to articulate. A potential bias is therefore present in the interpretation of the data, and without the counterbalance of the GPs’ viewpoints, there is a risk of overemphasizing patient-centric issues while potentially overlooking systemic or provider-side factors that influence the video consultation experience.

Second, and from a methodological standpoint, combining data deriving from different sub studies and generated by various interviewers in one analysis may present a limitation. While this approach has its strengths, e.g., allowing for a large and diverse sample, the subjective influence on the data of each interviewer becomes less transparent. However, the fact that we found that the data from each sub study, despite individual differences tied to each sub study, “rang true” in relation to an overarching theory, adds to the validity of the findings, demonstrating a degree of data triangulation that we view as a strength. Additionally, involving researchers from diverse disciplinary backgrounds in data collection and analysis also enhances the trustworthiness of the study results.

Third, the recruitment strategies of the sub studies may have introduced selection biases. There may have been an over- or underrepresentation of patients with certain experiences and opinions, e.g., patients that were particularly capable of articulating their views and experiences with video consultation or people being particularly satisfied or dissatisfied with the video-mediated interaction. In regards to the issue of over- or underrepresentation of patient experiences, one notable limitation of our study is the overrepresentation of patient accounts relating to mental health issues. While our dataset includes patients with various health issues, the accounts involving mental health - drawing on both actual and hypothetical experiences - provided the most explanatory power in our analysis. This is likely due to the perceived vulnerabilities and ethical stakes associated with mental health issues. As a result, these experiences may disproportionately have influenced our interpretations. Importantly, however, our key findings, relating to video consultations being experienced as more superficial, lacking in social talk, and characterized by a sense of transactional efficiency, were consistent across all physical and mental healt issues.

Finally, the patient experiences highlighted in this study are specific to the health care system and the general practice setting in Denmark. However, we believe that our findings are transferable to similar contexts, i.e., comparable health care systems, including general practice settings. Further research is needed that investigate whether the findings presented in this paper are transferable to psychotherapeutic settings where it may be the case that video-mediated presence and empathy are sensed to a higher degree and the face obscured to a lesser extent.

## Footnotes

1 Vidolov (2024), drawing on Merleau-Ponty’s perspective on intercorporeality, explains intercorporeality as: “the constitutive interconnection between bodies” (p. 244).

2 “Interaffectivity” signifies, according to Fuchs (2014), “the direct feedback from the embodied contact, based on emotional cues and expressive gestures by which we perceive one another empathetically” (p. 167). Fuchs thus argues that interaffectivity provides the basis for a primary empathic understanding of the other and that, if missing, empathic response to the other likely turns into projection of one’s own emotions to the other.
